# Defining the Role of Essential Genes in Human Disease

**DOI:** 10.1371/journal.pone.0027368

**Published:** 2011-11-11

**Authors:** Jonathan E. Dickerson, Ana Zhu, David L. Robertson, Kathryn E. Hentges

**Affiliations:** Faculty of Life Sciences, University of Manchester, Manchester, United Kingdom; The Centre for Research and Technology, Hellas, Greece

## Abstract

A greater understanding of the causes of human disease can come from identifying characteristics that are specific to disease genes. However, a full understanding of the contribution of essential genes to human disease is lacking, due to the premise that these genes tend to cause developmental abnormalities rather than adult disease. We tested the hypothesis that human orthologs of mouse essential genes are associated with a variety of human diseases, rather than only those related to miscarriage and birth defects. We segregated human disease genes according to whether the knockout phenotype of their mouse ortholog was lethal or viable, defining those with orthologs producing lethal knockouts as essential disease genes. We show that the human orthologs of mouse essential genes are associated with a wide spectrum of diseases affecting diverse physiological systems. Notably, human disease genes with essential mouse orthologs are over-represented among disease genes associated with cancer, suggesting links between adult cellular abnormalities and developmental functions. The proteins encoded by essential genes are highly connected in protein-protein interaction networks, which we find correlates with an over-representation of nuclear proteins amongst essential disease genes. Disease genes associated with essential orthologs also are more likely than those with non-essential orthologs to contribute to disease through an autosomal dominant inheritance pattern, suggesting that these diseases may actually result from semi-dominant mutant alleles. Overall, we have described attributes found in disease genes according to the essentiality status of their mouse orthologs. These findings demonstrate that disease genes do occupy highly connected positions in protein-protein interaction networks, and that due to the complexity of disease-associated alleles, essential genes cannot be ignored as candidates for causing diverse human diseases.

## Introduction

Much effort has been invested in identifying the set of genes that when mutated have a causal relationship with human disease. While many characteristics of genes associated with disease have been examined, prior studies have presumed that these disease genes form a homogeneous group sharing particular characteristics, distinct from non-disease genes [1], [2], [3], [4]. Further studies that classified disease genes based on their requirement during development, or essentiality, led to the conclusion that the majority of disease genes are non-essential [5], [6], [7], [8]. This conclusion is drawn from the analysis of human disease genes based on the phenotypes of their mouse orthologs. Disease genes whose mouse orthologs produce lethal phenotypes when deleted were considered essential, and all other genes considered non-essential. This classification, however, over-estimates the size of the non-essential gene group, due to the inclusion of genes with no reported knockout data. As currently only approximately 9% of mouse genes have been knocked out (Dataset S1), it is very likely that disease genes with no known mouse knockout phenotype would include both lethal and viable genes. Therefore, to include genes with no knockout data in the viable gene group confounds the analysis, and could lead to erroneous conclusions about the relative importance of lethal and viable genes in human disease.

It has also been proposed that mutations in the human orthologs of essential mouse genes will cause lethality in human pregnancies, accounting for spontaneous miscarriages [5], [9]. The authors of one study therefore conclude that essential genes are not human disease genes [9], as mutations prevent viability. However, this assumption fails to consider the impact of alleles on gene function. Genes have been defined as essential due to the phenotype of mouse knockouts, which result from a deletion of the protein-coding region of the gene from the genome. These mouse mutants therefore are null alleles, and represent the phenotype caused by complete absence of functional protein. However, point mutations in these same genes do not necessarily fully remove protein function. Gene alleles with reduced function, called hypomorphic alleles, therefore can generate different phenotypes from those of null alleles. Although null or severe loss-of-function mutations in essential genes may indeed contribute to spontaneous miscarriages, hypomorphic mutations in the same genes can contribute to less severe abnormalities that are recognized as human disease. Therefore, the human orthologs of genes required for embryonic development in the mouse can cause disease in mutated forms through a variety of mechanisms. For example, some orthologs of mouse essential genes cause human congenital birth defects in a manner that resembles their mouse knockout phenotypes [10], [11], [12], [13]. Other orthologs of mouse essential genes show haploinsufficiency in the human, such that they cause an abnormal phenotype in the heterozygous state [14], [15], [16], [17]. Embryonic lethal mouse genes can also have disease-associated orthologs in the human due to the presence of hypomorphic mutations in the human population, which represent a less severe loss of gene function than that observed in the mouse knockouts [18], [19], [20]. Alternatively, embryonic lethal genes can also undergo gain-of-function mutations, causing over-expression or increased activity, which contribute to human disease in a manner different from their mouse loss-of-function phenotype [21], [22], [23].

Given that several genes known to cause lethality in mouse knockouts also cause human disease due to point mutations or genomic rearrangements, we hypothesise that essential genes form an important group of disease genes that will have different characteristics from non-essential disease genes. As mouse targeted deletions provide a source of experimental analysis of null alleles, we used data on lethal and viable mouse knockouts as a proxy for human essential genes. The similar physiology and genome structure between the mouse and human facilitate ortholog comparison and functional identification between the two species. To determine if differences exist between essential and non-essential disease genes we examined several parameters in our analyses, including the physiological systems affected by each disease gene, the connectivity of each gene in protein-protein interaction networks, and the genetic mechanisms by which genes cause human disease.

Our results demonstrate that essential and non-essential disease genes have a tendency to differ in the types of disease they cause, the mode of disease inheritance, and the number of protein-protein interactions in which they participate. We find that essential disease genes comprise a major portion of disease genes, and are associated with many types of human diseases affecting diverse physiological systems. Additionally, non-essential disease genes form a distinct class to essential disease genes for nearly every parameter examined, and are also not similar in characteristics to non-disease genes. We conclude that disease genes cannot be considered a homogeneous group of genes, and that gene essentiality is an important determinant of disease type.

## Results

### Classification of disease genes

We identified 1,965 human disease genes from OMIM's morbid map [24], [25]. To assess whether essentiality was correlated with particular disease gene properties, we grouped the disease genes into viable and lethal categories, based on inference from mouse knockout data [26], [27]. Approximately 40% of human disease genes (793/1965) had a knockout reported for their mouse ortholog. We term human disease genes with essential mouse orthologs (those with lethal knockout phenotypes) as “disease lethal” (DL, n = 673) genes and those with non-essential mouse orthologs (those with viable knockout phenotypes) as “disease viable” (DV, n = 120) genes. It is important to note that the essentiality classification is based upon mouse null alleles, whereas human disease alleles are rarely null mutations. Therefore, when a gene is referred to as “disease lethal” it is not an indication that the human diseases associated with mutations in that gene are lethal, but rather that complete removal of protein function causes lethality in mouse. Importantly, in contrast to prior studies [5], we considered all disease genes for which there is no mouse knockout data available as a separate group of “unknown” disease genes (DU, n = 1172). These gene groups and classifications were used in all subsequent analyses (Table 1, Dataset S1).

**Table 1:** Summary of the number of genes of each category characterized for each parameter examined.

	Viable	Lethal	Disease Viable	Disease Lethal	Disease Unknown	All Disease
**Number of genes**	672	1299	120	673	1172	1965
**Number of genes with protein-protein interactions**	489	1093	90	310	924	1324
**Number of genes with gene ontology annotations**	670	1288	119	670	1166	1955
**Number of genes with disease class annotations**	-	-	68	214	830	1112
**Number of genes with disease mode of inheritance classifications**	-	-	64	185	626	875
**Number of genes with disease gain/loss of function classifications**	-	-	73	219	987	1279

Data was not available for all genes for each parameter. Some analyses were performed only for disease gene datasets, such as disease classification, and others also evaluated for the viable and lethal datasets.

Notably, for those human disease genes with a known mouse knockout phenotype, orthologs of essential mouse genes are more highly represented (673/793, 85%) than orthologs of non-essential mouse genes (120/793, 15%), a finding that contradicts prior studies [5], [6], [7], [8]; removing the “unknown” class (DU) has a dramatic impact on the analysis of disease genes. It has been reported that the published mouse knockout dataset is enriched for developmental genes [28]. The percentage of total mouse knockouts with a lethal phenotype is 66% (1299/1971). Yet for disease genes with known mouse essentiality status, lethal genes comprise 85% of the dataset (673/793; Table 1, Dataset S1, χ2 p<0.05). Because the proportion of disease genes with essential mouse orthologs does not simply reflect the relative proportions of reported lethal and viable knockouts, we conclude that experimental bias cannot solely explain the abundance of essential gene orthologs among human disease genes (Dataset S1, χ2 p<0.05).

We expect that additional essential genes will be found in the DU dataset. For example, genes required for basal cellular functions, or “housekeeping genes”, have been proposed to form a subgroup of essential human genes [29]. Therefore, we quantified the percentage of housekeeping genes (from reference 30) in our datasets as compared to the entire human genome (Dataset S2). We find that 5.5% of the genes in the DU dataset (64/1172) have been identified as housekeeping genes, while the percentage of housekeeping genes overall in the human genome is only 2.5% (609/24789) [30]. This difference is statistically significant (DU genes vs not DU genes by housekeeping genes vs not housekeeping genes, Dataset S1, χ2 p<0.05). Additionally, as part of a large-scale effort to generate targeted deletions in all mouse genes, an initial analysis of 355 new mouse knockout lines has revealed that approximately 30% exhibit embryonic lethality (http://www.sanger.ac.uk/mouseportal/). Of the new knockouts generated, we found that 22 are mouse orthologs of human DU genes. Of these, 6 exhibit lethal phenotypes (http://www.sanger.ac.uk/mouseportal/), confirming that there are DL genes in the DU dataset, which were misclassified as DV in prior studies [5]. Due to the under-representation of housekeeping genes in mouse knockout experiments and the evidence of new knockouts with lethal phenotypes, we infer that there are additional essential genes in the DU dataset, the presence of which, when properly annotated, would increase the overall number of disease genes with essential functions.

The gene conservation between mouse and human orthologs of disease genes has been assessed with respect to essentiality, including a quantification of the frequency of orthologs of human disease genes among genes with no phenotype, non-lethal phenotypes, or lethal phenotypes in mouse knockout experiments [9]. Park et al. found that human orthologs of the lethal gene group had the most complete mapping to human disease genes, although they did not differ significantly from the percentage of non-lethal genes that were associated with disease [9]. However, rather than determining the distribution of disease genes among mouse knockout groups, in our work we have performed the opposite analysis to determine the prevalence of lethal and viable mouse knockout gene orthologs among all disease genes. We find that 34% of disease genes have a mouse ortholog with a lethal phenotype in knockout experiments (673/1965), while only 6% of disease genes (120/1965) have a mouse ortholog with a viable phenotype. Thus, a greater proportion of human disease genes are orthologous to an essential mouse gene.

### Essentiality affects disease gene classification by physiological system

**Figure 1:**
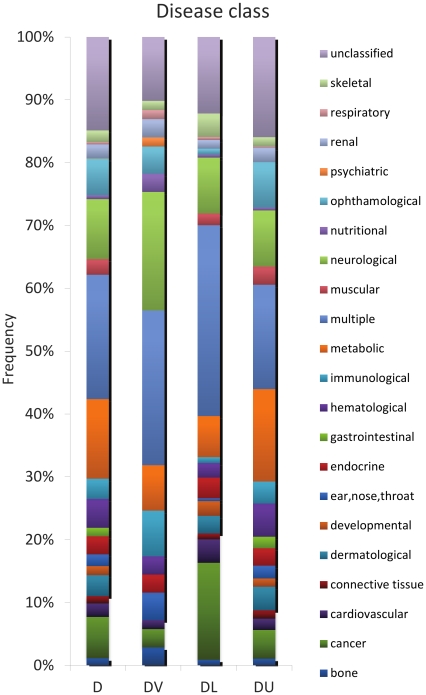
Physiological system analysis of disease genes. Distribution of all Disease genes (D), Disease Viable genes (DV) and Disease Lethal genes (DL) in different disease classes, according to the physiological system affected. The D set corresponds to all disease genes without separation according to essentiality.

Essential genes have been considered non-disease genes by others due to their presumed role in developmental defects and associated lethality [7], [8], [9]. Since, on the contrary, we find that the majority of disease genes with known essentiality status were lethal genes, we next sought to examine the diseases with which lethal genes are associated. Using MeSH classifications for disease genes, we identified the disease types associated with each disease gene. When grouped according to essentiality, disease genes differ in the specific processes that they disrupt (Figure 1, for all DU data see Figure S1). Thirty percent of disease lethal genes are associated with diseases affecting more than one tissue, as compared to 19% of all disease genes, which is a significant over-representation (Fisher's exact test p<0.05, Dataset S3), indicating that multiple physiological systems are affected by disease mutations in essential genes. DL genes, as opposed to DV or DU genes, are also highly associated with multiple types of cancer (15% of DL genes compared to 7% of total disease genes, Fisher's exact test p<0.05), which may be explained by the observation that targeted deletions of cancer associated gene orthologs in mice often reveal a requirement for those genes in embryonic development, while the gene mutations observed in humans are hypomorphic alleles, activating mutations, or mutations in somatic cells [31]. Despite a known developmental role for their mouse orthologs, DL genes were not significantly over-represented in developmental processes (2% of DL genes) as compared to all disease genes (1% total disease genes) in this analysis. Overall, we find that DL genes are associated with a variety of diseases affecting nearly all physiological processes, and are not restricted to causing developmental defects.

### Disease lethal genes tend to be highly connected in protein-protein interaction networks, while disease viable genes are less connected

**Figure 2:**
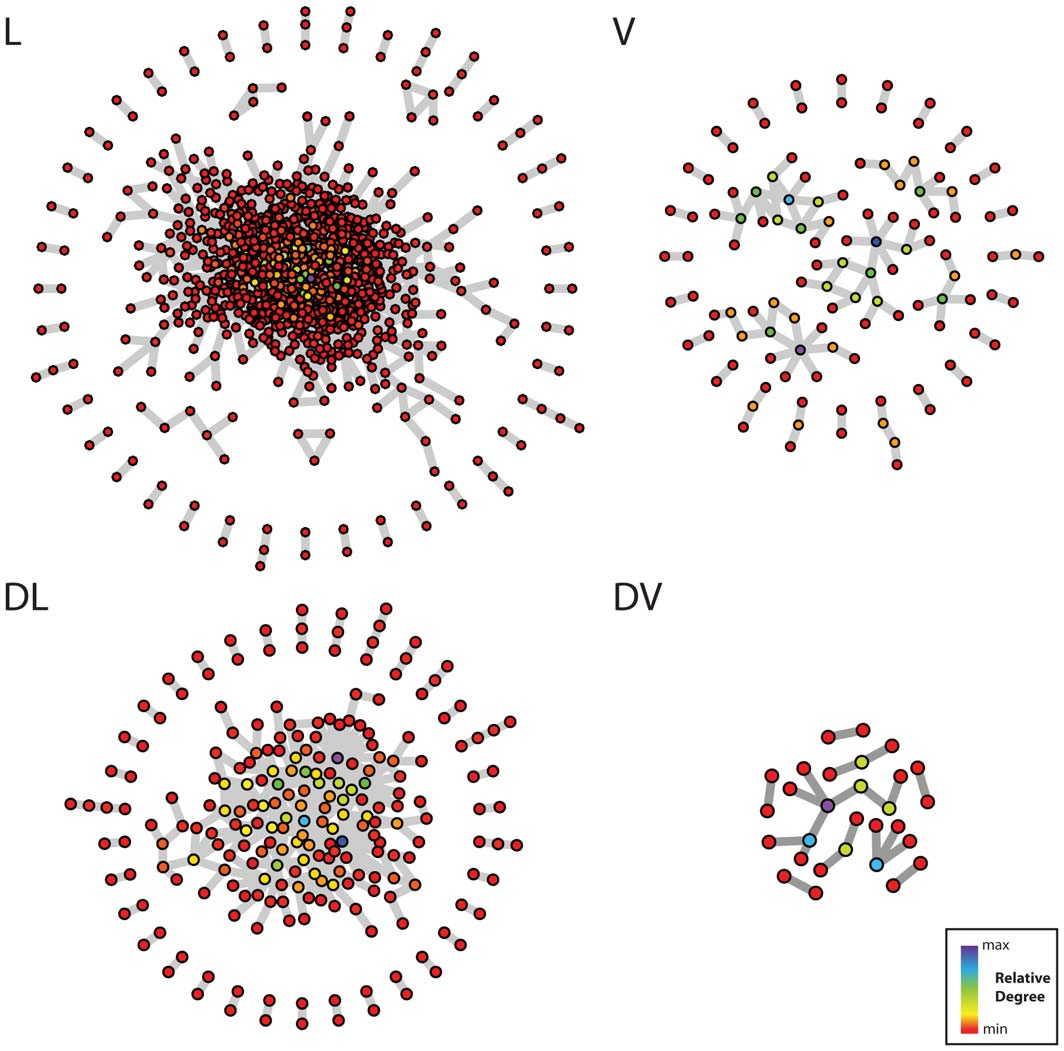
Network representation of protein-protein interaction between proteins chosen from the viable (V), lethal (L), disease viable (DV) and disease lethal (DL). For clarity, interactions are only displayed in the figure if both interacting partners have the same classification (e.g. DV-DV interactions). However, statistical analysis (Table 2) was performed for all interactions (e.g. proteins of the same classification interacting and proteins of different classifications interacting). The color corresponds to node degree (relative to each network) as indicated for each panel, with the lowest degrees in red and highest degrees in purple. The node degree denotes the number of PPIs for a given gene.

Rarely are biological functions attributed to a single molecule; instead, all components of a cell are intrinsically related and can be thought of as a network of interacting modules. Genes that are highly connected in protein-protein interaction (PPI) networks may therefore affect multiple biological processes when mutated, due to disruption of interactions with a variety of other genes in the network. Based on the finding that DL genes are highly represented in diseases affecting multiple physiological systems, we examined the connectivity of proteins encoded by different types of disease genes in PPI networks. Previous studies have failed to distinguish between proteins encoded by genes of unknown essentiality and those encoded by genes that are non-essential [5]. This fundamental gene classification difference influences the conclusions drawn from the resulting PPI networks. Accordingly, we separately considered essential, non-essential and unknown essentiality disease proteins for PPI network analysis (Figure 2). Our results show that DL proteins have a higher average degree (more interactions for each protein) than DV and V proteins (Table 2). We found that L proteins have the highest maximal degree (138) and average degree (3.9) when compared to all other groups, indicating that L proteins have many interactions. The variation in connectivity among different gene classes is not simply a function of the number of proteins in each class, as the ratio of nodes to interactions varies for each gene group (Dataset S4, χ2 p<0.05).

**Table 2:** Protein-Protein Interaction network properties of disease genes.

						Largest Connected Component		
	Proteins	Inter-actions	Max Degree	Avg Degree	Comps	Proteins	Inter-actions	Avg Degree
Disease Lethal	1943	2778	104	2.9	70	91%	96%	3.0
Disease Viable	1535	1600	92	2.1	151	71%	81%	2.4
Lethal	3638	7083	138	3.9	86	94%	98%	4.1
Viable	1436	1654	71	2.3	86	84%	91%	2.5
Disease Un-known	5116	8918	92	3.5	224	89%	96%	3.8

Data for PPI networks for disease lethal (DL), disease viable (DV), lethal (L), viable (V) and disease unknown (DU) genes. “Proteins” represent the sum of proteins in a given group (DL, DV, L, V or DU) and their interacting partners (from any group). Statistical analysis was performed for all interactions (e.g. proteins of the same classification and proteins of different classifications). The total number of PPIs are indicated under “Interactions” and the number of PPIs for the most highly connected protein is indicated under “MaxDegree”. “Average Degree” corresponds to the mean number of PPIs for all interacting proteins. “Comps” designates the number of independent groups (components) of interactions, the largest of which comprises the “Largest Connected Component”. The percentages of proteins and interactions, and the average degree found within the largest connected component are given.

Our analysis of the PPI for proteins with unknown essentiality suggests that the DU group contains a mixture of essential and non-essential proteins. The DU network (consisting of interactions between DU proteins and those of any other group) contains the largest number of individual components (224), yet the majority of the proteins (89%) and interactions (96%) are within the largest connected component. Therefore, there are many small components with few proteins and interactions on the periphery of the DU network, which may represent the non-essential proteins within the DU group. We find that DU proteins participate in a similar number of interactions to L proteins (Average Degree, Table 2). The degree distributions (Table 2) verify the DU network is more similar to the DL network in connectivity (Wilcoxon–Mann–Whitney and Kolmogorov–Smirnov tests, both p<0.05), than to the DV network. The outlying data points in the prior analysis of viable disease gene PPI networks [5] presumably reflect the inclusion of essential genes that have no mouse knockout (that we classify as DU) in their viable gene dataset (Table 2).

### Disease proteins segregate to different cellular compartments based on essentiality

**Figure 3:**
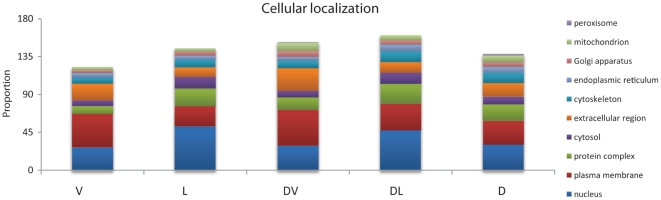
GO analysis of disease genes. Distribution of Viable (V), Lethal (L), Disease Viable (DV), Disease Lethal (DL), and all disease (D) proteins analysed for cellular localization according to GO terms.

Interactions between proteins can only occur when those proteins are co-localized. In yeast, it has been shown that sub-cellular localization affects the degree of network interactions, with nuclear proteins being more highly connected than those in the cell periphery [32]. As we found differences in the degrees of interactions for DV and DL proteins, we tested whether this was a result of differences in the sub-cellular localization of the proteins. Using Gene Ontology (GO) annotations [GO, 33], we found that viable and lethal genes vary in the cellular compartments to which they are localized (Figure 3). Both DL genes and L genes, which are highly connected, are statistically overrepresented in the nucleus (46.45% and 50.8% respectively, see Dataset S5 for statistical analysis data, and Text File S1 for a description of statistical data files). In contrast, DV genes, in addition to V genes generally, are enriched for localization to the plasma membrane (Fisher's exact test p<0.05). However, DV genes are also statistically overrepresented in the extracellular region (Fisher's exact test p<0.05). Our findings on sub-cellular localization are in agreement with those for yeast proteins [32], indicating that DL genes may show a greater number of PPIs due to their higher probability of localization within the nucleus. In accordance with differences in the subcellular localization of DL and DV genes, differences in molecular function and biological process are also detected between groups according to GO annotations (Figure S2).

### Essentiality does not affect disease mechanism

**Figure 4:**
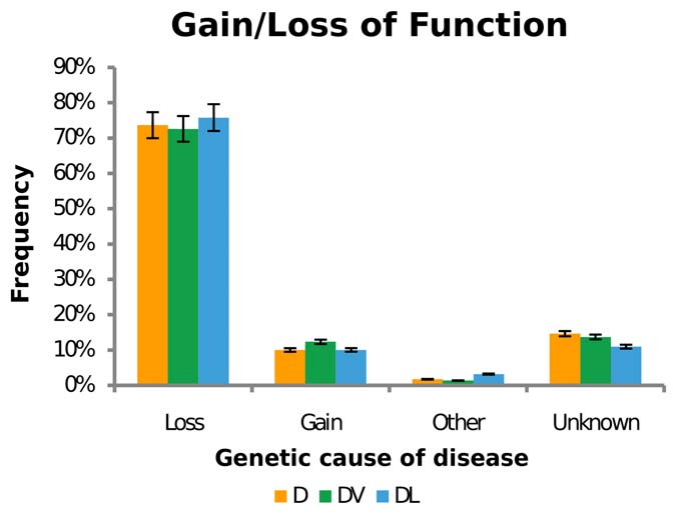
Disease mechanism analysis. Classification of disease mechanism in the total disease gene set (D, red bars), and Disease Viable gene (DV, green bars) and Disease Lethal gene (DL, blue bars) subsets. Other refers to diseases caused by chromosomal translocations or chimeric proteins.

Genetic alterations that contribute to disease can manifest as a result of loss of protein function, gain of a new or enhanced/dysregulated protein function, or be a consequence of large chromosome or gene rearrangements, such as translocations that generate chimaeric proteins (classified as “other”). We used the characterization of disease mutations in OMIM [24] to classify such functional changes for disease genes. Our results show that essentiality does not appear to correlate with the propensity of a disease to be originated by gain or loss-of-function mutations in a gene. Independent of essentiality, the majority of diseases arise from loss-of-function mechanisms (around 70%) and only a small number are caused by a gain-of-function mutations (around 10%), or chromosomal translocations (Figure 4, Dataset S5). However, it is possible that these results are due to limited data availability or our classification methods. Furthermore, the low proportion of inherited diseases associated with translocations is likely due to infertility associated with abnormal karyotypes.

### Disease lethal genes are more likely to demonstrate a dominant mode of inheritance than other disease classes

**Figure 5:**
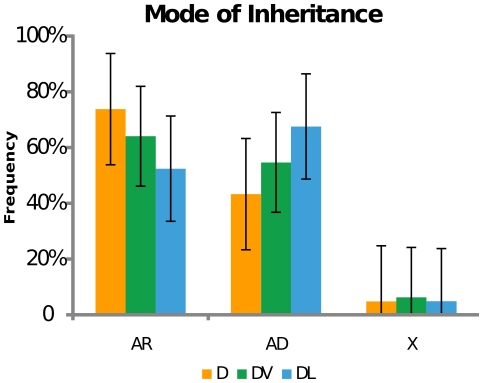
Mode of inheritance of disease genes. Proportion of disease genes inherited in an autosomal dominant pattern (AD), autosomal recessive pattern (AR) or X-lined pattern (X) in the total Disease gene set (D, red bars), or Disease Viable gene (DV, green bars) and Disease Lethal gene subsets (DL, blue bars).

Although genes from all groups seemingly cause disease predominantly through loss-of-function mechanisms, it is possible that essentiality may affect the mode of inheritance of disease genes. We therefore classified disease gene mutations as autosomal dominant, autosomal recessive, or sex-linked, and categorized them according to essentiality. Importantly, for this study we included any mutant allele with a described mode of inheritance, so a particular gene could be included in both the autosomal dominant and autosomal recessive categories if it had different mutations exhibiting those inheritance patterns. The frequency of disease mutations with sex-linked inheritance is below 10% for all of the groups (Figure 5). It was observed that the DL gene set showed a higher proportion of autosomal dominant mutations than autosomal recessive (Fisher's exact test p<0.05, Dataset S5). The high representation of dominant inheritance patterns in essential genes may be reflective of actual semi-dominant mutations, where disease represents a heterozygous phenotype, and lethality a homozygous phenotype.

## Discussion

To refine understanding of the properties of human disease genes, we classified disease genes as essential or non-essential, and assessed whether these two types of disease genes have specific attributes for a variety of parameters. We also considered those disease genes with unknown essentiality as a separate group. We propose that this separation allows for an improved understanding of the features of human disease genes, building upon the finding that disease genes with essential and non-essential orthologs differ in nearly every parameter for which they were studied. Our findings are based on the current annotations of gene phenotypes in the mouse knockout literature. Although in some cases mouse knockouts of human disease genes have demonstrated phenotypes that are not readily comparable to the human disease state [34], the inability to assess essentiality in the human necessitates inferring essentiality from other organisms. The mouse is clearly the most similar model organism for which there is essentiality data. Furthermore, large-scale mouse knockout projects will in future provide additional data on essentiality for consideration [35].

Separating disease genes according to their essentiality status provides insights that expand upon observations from prior studies on disease gene PPI networks [5], [36], [37]. Our results agree with prior studies that reported a central position for DL genes in PPI networks [5], [37]. However, using our classification system for disease genes where those of unknown essentiality are classified as a separate group, we find that more disease genes can be classified as essential rather than non-essential. Therefore, our interpretation of the PPI network analysis differs from prior studies. Mainly, as DL genes constitute the majority of disease genes, and DL genes have multiple PPIs acting as hubs in the PPI network, we conclude that it is very likely that disease genes are found at highly connected central positions in PPI networks.

At the molecular level, disease genes are segregated to different cellular regions when considered in the context of essentiality. The number of PPIs differs for the DL and DV gene groups, although that may be due to differences in sub-cellular localization. DL genes are both found to have more interactions and to be more likely to be localized to the nucleus. It has been demonstrated that nuclear proteins have higher numbers of interaction partners in PPI networks [32], which may explain the basis for the difference in connectivity between DL and DV genes.

Prior studies have suggested that essential genes contribute to human disease by causing spontaneous miscarriages and birth defects [5], [7], [8], [9], [37]. While this conclusion is reasonable for the null alleles of DL genes, we conclude (from our analysis of the physiological systems affected by disease genes) that DL genes also contribute to adult disease in humans due to additional disease alleles that do not represent functional null alleles. Therefore, the definition of essentiality needs to be precisely applied to null alleles. A consideration that other alleles of essential genes, such as hypomorphic alleles, may not have lethal phenotypes needs to be incorporated into bioinformatic studies of disease genes. Indeed our study reveals that DL genes are more highly associated with diseases that affect multiple physiological systems. This finding suggests that disease lethal genes have pleiotrophic functions. For example, many house-keeping genes are found within the lethal group, and these genes are likely to function in many or all cell types. Previous studies have suggested that house-keeping genes, defined as genes with ubiquitous expression patterns, are essential for organism survival [29] and, as a consequence, ‘mild’ mutations in these genes will cause diseases with symptoms in several tissues. An analysis of ubiquitously expressed genes as compared to human disease genes revealed that the two classes of genes differed in evolutionary and functional properties [29]. However, ubiquitous expression is not a comprehensive indicator of essentiality, and genes with housekeeping functions may not necessarily have lethal knockout phenotypes. Our work, thus, demonstrates that when essentiality, as inferred by mouse knockout phenotypes, is considered explicitly, disease genes themselves display differing characteristics based on their essentiality status.

Interestingly, DL genes were found to be over-represented in cancer. Many oncogenes are associated with cell proliferation and death mechanisms. While disruption of these processes during embryonic development would likely prevent the survival of the organism, cancers commonly result from somatic cell mutations disrupting normal controls of the cell cycle [31]. Indeed, cancer can be viewed as a developmental disease [38], [39], [40], because developmental genes promoting cell proliferation become reactivated in the adult and drive proliferation in an uncontrolled fashion. A bioinformatic prediction strategy for identifying cancer genes has been developed, although the role of essential genes has not explicitly been incorporated into this model [41]. Our results suggest that identifying essential genes may further refine the prediction of genes likely to be associated with causing cancer. DV genes primarily affect systems that are not required for basic survival of the organism. For example, while a high percentage of disease viable genes are associated with psychiatric and immune system diseases, they are under-represented among cardiovascular diseases.

We also detected a difference in the mode of inheritance of disease genes when classified according to essentiality. We find that disease lethal genes are more likely to demonstrate an autosomal dominant mode of inheritance. This reflects the tendency for disease to occur in the heterozygous state in these individuals, and that homozygosity for disease mutations would present a more severe phenotype causing lethality. Human disease mutations are not often nulls, and essentiality and disease can be considered on a spectrum with respect to mutant alleles. While severe mutant alleles that eliminate protein function represent the null state and confer lethality, hypomorphic alleles simply reduce protein function below an optimal level, resulting in a phenotype recognized as disease. In this manner, lethal genes can have mutations that only reduce protein function and therefore present as disease in the human population, allowing for inheritance of these more mild alleles in genetic diseases.

From our study we can now present a composite profile of human disease gene types. We have found that DL and DV genes differ from each other in many of the characteristics we have analyzed. Overall, DL genes are more likely to be involved in more protein-protein interactions, encode nuclear proteins, be associated with diseases such as cancer and those affecting multiple systems, and have an autosomal dominant mode of inheritance. In contrast, DV genes have a tendency to localize to the plasma membrane or extracellular regions, be involved in neurological or immune system diseases, and have an autosomal recessive mode of inheritance.

We have shown that disease genes are not a homogenous group and should be considered in the context of the functional importance of the gene with which they are associated. Moreover, in contrast to prior studies [5], [6], we find that when disease genes with unknown essentiality are considered as a separate group from non-essential disease genes, the majority of disease genes are essential. We propose that rather than solely contributing to spontaneous miscarriages or birth defects due to severe loss of function mutations, as has been stated in prior studies [7], [8], [9], DL genes have a variety of disease associated alleles that represent a spectrum of human diseases affecting both development and adult physiological systems. The recognition that disease genes are not a homogenous subset of human genes, and that essential genes cannot be excluded from consideration as candidates for all types of human disease genes, will aid in the identification of candidate disease gene loci for a variety of human diseases.

## Materials and Methods

### Data retrieval

We obtained the human–mouse orthology and mouse viable/lethal phenotype data from Mouse Genome Informatics (http://www.informatics.jax.org) [26], to give 2,360 human genes with information about lethality status of the mouse knockout of their ortholog in the whole genome, without consideration of whether the gene is annotated as a human disease gene. These were verified manually by checking phenotypes using PubMed. We considered the annotations of embryonic, postnatal, prenatal and perinatal lethality as lethal phenotypes. These data are an appropriate proxy for gene essentiality in humans and are herein mentioned as viable and lethal. After removing redundancy, we find 1,299 and 672 human orthologs of mouse lethal and viable genes respectively. Only mouse genes with known phenotypes resulting from targeted deletions (knockouts) were included in the study.

To create the human protein-protein interaction network, encoded proteins for each gene were determined from the Entrez gene database and interactions were derived from multiple sources: BioGRID (http://www.thebiogrid.org) [42], BIND (http://www.bind.ca) [43] and HPRD (http://www.hprd.org) [44] and filtered from the NCBI “interactions” file (ftp://ftp.ncbi.nlm.nih.gov/gene/GeneRIF). Interaction data contained in these datasets are derived from multiple sources, such as Y2H, co-imunoprecipitation and so on. The resulting protein-protein interaction network consisted of 8,880 nodes (proteins) with 33,979 edges (interactions).

A dataset of 1,965 disease genes was retrieved from the OMIM database [24], and cross-referenced with OMIM's morbid map to provide disease-gene-phenotype relationships [25]. Of these, 1,324 of the disease genes were present in the protein-protein network; 90 of which could be found in the viable network and 310 in the lethal network.

From Eisenberg and Levanon [30], we obtained 600 housekeeping genes and converted the given nucleotide accessions to gene loci names and Entrez accession numbers using the NCBI Entrez gene database.

### Disease classification

The classification of the different disease genes into their corresponding disease categories was based on the Medical Subject Headings controlled vocabulary (MeSH; http://www.nlm.nih.gov/mesh/meshhome.html) [45] as previously described [46]. MeSH hierarchically describes diseases (in addition to other life science categories), e.g., diseases to digestive system diseases to digestive system neoplasms and so on. High level terms were combined with classifications from Goh [5] to provide a consistent annotation of disease as in [46].

### Network connectivity and centrality measurements

Cytoscape (version 2.62) [47] and Navigator (version 2.1.13) [48] were used to visualize the protein-protein network and to analyse the number of highly connected proteins (hubs) and the number of hub-hub connections in the network belonging to each dataset. R [49], using the igraph package [50], was used to verify network properties for each subset of nodes. Degree is defined as the the total number of edges (interactions) incident upon a node (protein), the distribution of which gives a probability distribution of degrees over the whole network. Components are the number of maximally connected independent groups of interactions, the largest of which is the largest connected component. Self-edges were ignored throughout. Quantitative analyses include all interactions where one (or both) of the partners is a member of the category (DL, DV, L, V or DU, Table 2). For Figure 2, to improve image clarity, only interactions observed between like-category partners (e.g. DV-DV) are visualized in a force-directed layout.

### Protein cellular localization, function and processes

Gene Ontology (GO) annotations for human genes were retrieved using the BINGO 2.3 plugin [51] present in Cytoscape [47] and from GO directly (http://www.geneonotology.org/GO.downloads.ontology.shtml) [33]. GO slim corresponds to a higher-level version of GO ontologies, that contains a subset of terms representative of the complete GO and were also downloaded from GO (http://www.geneontology.org/GO.slims.shtml). Functional analysis corresponds to gene ontology terms from the molecular function category that have the term "activity" in their name.

### Disease Mechanism

We exploited the rich annotation of OMIM to classify diseases as resulting from gain-of-function or loss-of-function mechanisms. Mutations that cause the formation of chimeric proteins due to translocations are included in a further ‘other’ category. For each OMIM record an automated simplistic word scoring process was used, whereby discriminating words and word stems, e.g. “deficiency”, “neomorphic”, “activation”, correspond to each category (gain, loss, neutral). The type of mutation was thus chosen according to the category that presented the highest word score. To verify this data, a separate Bayesian classifying approach was used to exclude method bias and error using Bishop for Ruby (http://bishop.rubyforge.org/), based on the Reverend classifier for Python (http://divmod.org/trac/wiki/DivmodReverend). OMIM records known to represent gain, loss or neutral consequences were manually selected and used as training sets. Frequent words were excluded from training and classification, as were non discriminating words common to each grouping. For example, words shared by a gain record and a loss record in the training set were excluded from our classification.

A separation of monogenic diseases from polygenic diseases was also performed (Table 3). The results with and without the polygenic diseases were similar. The group without the polygenic diseases is presented.

**Table 3:** Number of genes associated with monogenic or polygenic diseases falling into each disease gene classification.

	D	DV	DL	DU
**Monogenic disease gene**	**1279**	**73**	**219**	**987**
**Polygenic disease gene**	**223**	**19**	**71**	**133**

### Mode of inheritance

The separation of the disease essential, disease non-essential, and disease unknown genes into the different modes of inheritance (autosomal dominant, autosomal recessive and sex-linked) was based on the categorization provided by Blekhman [52].

### Statistical analyses

Statistical analyses were performed throughout in R [49]. P-values were calculated using Fisher's exact, Wilcoxon–Mann–Whitney or Kolmogorov–Smirnov tests as indicated. The Benjamin & Hochberg False Discovery Rate (FDR) was used to calculate corrected p-values [53].

## Supporting Information

Figure S1 **Disease Unknown gene analysis for each parameter.** Panels A-H correspond to main body figures: protein-protein interactions degree distribution (A) and network representation (B); cellular localization (C), molecular function (D) and biological processes (E) distributions; disease classes distribution (F); disease mechanism (G); disease gene inheritance (H).
(EPS)

Figure S2 **Analysis of GO terms describing molecular function and biological process.** Distribution of Viable (V), Lethal (L), Disease Viable (DV), Disease Lethal (DL), and all disease (D) proteins analysed for molecular function binding (Panel A), molecular function (Panel B) and biological process (Panel C) according to GO terms.
(PDF)

Dataset S1 **Examples of statistical calcuations and lists of classification of lethal, viable, and disease genes.**
(XLS)

Dataset S2 **List of housekeeping genes in each category.**
(XLS)

Dataset S3 **Disease gene classification according to disease type.**
(XLSX)

Dataset S4 **Analysis of network data.**
(XLS)

Dataset S5 **Statistical test data.**
(XLS)

Text File S1 **Legend to Dataset S5.**
(DOC)
