# Supplementary figures and images for: Defining the Role of Essential Genes in Human Disease
Source: PLoS One. 2011 Nov 11;6(11):e27368. doi: 10.1371/journal.pone.0027368 (PMC3214036; doi:10.1371/journal.pone.0027368)

A

## Molecular function (binding)

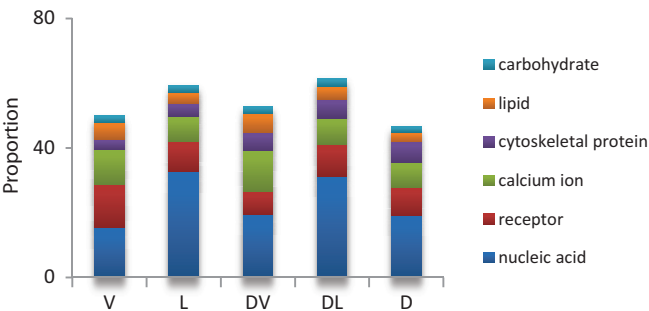

B

## Molecular function

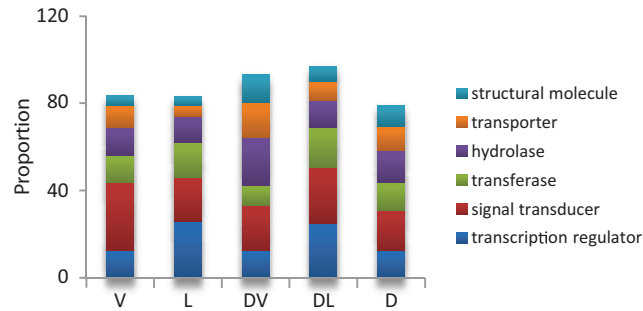

C

## Biological process

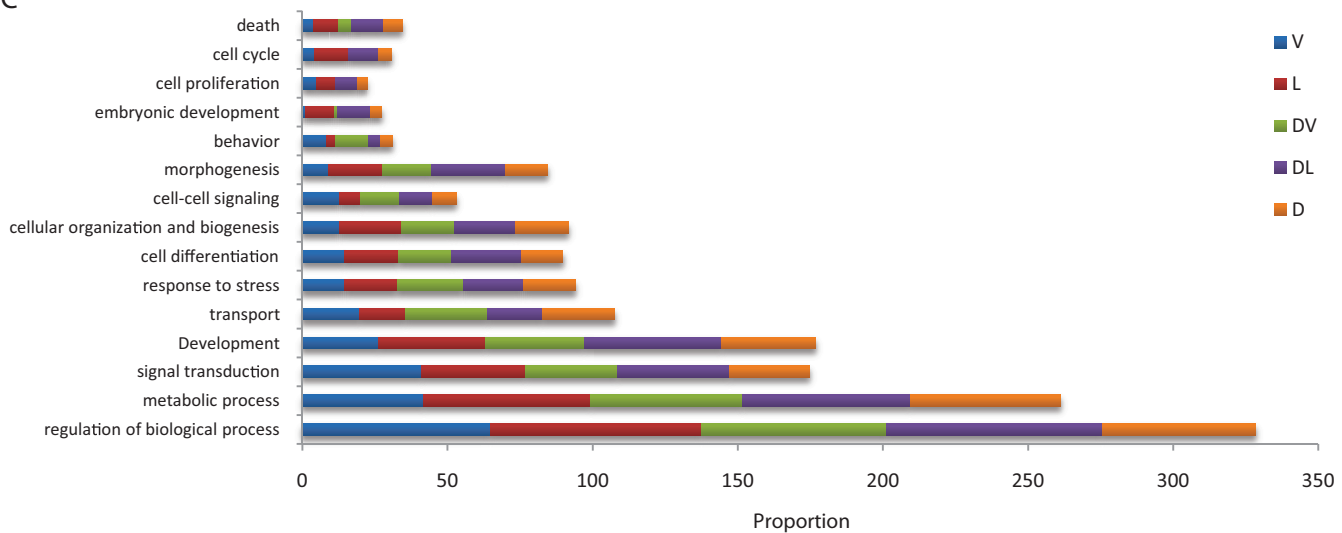

Supplement: Figure S2 — Analysis of GO terms describing molecular function and biological process. Distribution of Viable (V), Lethal (L), Disease Viable (DV), Disease Lethal (DL), and all disease (D) proteins analysed for molecular function binding (Panel A), molecular function (Panel B) and biological process (Panel C) according to GO terms. (PDF) [file pone.0027368.s002.pdf]
