# Supplementary material for: Defining the Role of Essential Genes in Human Disease
Source: PLoS One. 2011 Nov 11;6(11):e27368. doi: 10.1371/journal.pone.0027368 (PMC3214036; doi:10.1371/journal.pone.0027368)
Supplement: Text File S1 — Legend to Dataset S5. (DOC) [file pone.0027368.s008.doc]

Description of worksheets in Dataset S5

**Stat_1** Wilcox test comparing the viable (V), lethal (L), disease viable (DV), disease lethal (DL) and disease unknown (DU) subsets Degree, considering significant differences when p-value is lower than 0.05. The first value in each cell is the Wilcoxon rank sum test statistic (W) and the second value is the p-value.

**Stat_2** Statistical analysis of GO terms associated with viable dataset using a hyper-

geometric test and calculation of the adjusted p-value (p-value corr.) through the

FDR method. X is the number of genes from the list associated with a particular

GO-ID, T is the number of genes from the genome associated with that GO-ID.

**Stat_3** Statistical analysis of GO terms associated with lethal dataset using a hyper-

geometric test and calculation of the adjusted p-value (p-value corr.) through the

FDR method. X is the number of genes from the list associated with a particular

GO-ID, T is the number of genes from the genome associated with that GO-ID.

**Stat_4** Statistical analysis of GO terms associated with disease viable dataset using a hypergeometric test and calculation of the adjusted p-value (p-value corr.) through the

FDR method. X is the number of genes from the list associated with a particular

GO-ID, T is the number of genes from the genome associated with that GO-ID.

**Stat_5** Statistical analysis of GO terms associated with disease lethal dataset using a hypergeometric test and calculation of the adjusted p-value (p-value corr.) through the

FDR method. X is the number of genes from the list associated with a particular

GO-ID, T is the number of genes from the genome associated with that GO-ID.

**Stat_6** Statistical analysis of GO terms associated with disease unknown dataset using a hypergeometric test and calculation of the adjusted p-value (p-value corr.) through the

FDR method. X is the number of genes from the list associated with a particular

GO-ID, T is the number of genes from the genome associated with that GO-ID.

**Stat_7** Statistical analysis of GO terms associated with disease dataset using a hypergeometric test and calculation of the adjusted p-value (p-value corr.) through the

FDR method. X is the number of genes from the list associated with a particular

GO-ID, T is the number of genes from the genome associated with that GO-ID.

**Stat_8** Statistical analysis of the disease class associated with disease viable using a Fisher test and p-value through FDR method (p-value corr.). x is the number of genes from disease viable within each disease class and Non x is the number of genes not from disease viable in each class.

**Stat_9** Statistical analysis of the disease class associated with disease lethal using a Fisher test and p-value through FDR method (p-value corr.). x is the number of genes from disease lethal within each disease class and Non x is the number of genes not from disease lethal in each class.

**Stat_10** Statistical analysis of the disease class associated with disease unknown using a Fisher test and p-value through FDR method (p-value corr.). x is the number of genes from disease unknown within each disease class and Non x is the number of genes not from disease unknown in each class.

**Stat_11** Binomial test to compare the proportion of gain and loss of function and others in proteins from the Disease Viable (DV), Disease Lethal (DL) and Disease Unknown (DU) subset. In each cell the first element is the Pearson Chi Square and the second is the p-value.

**Stat_12** Fisher test to compare the proportion of proteins caused by each inheritance mode in Disease Viable (DV), Disease Lethal (DL) and Disease Unknown (DU) subset. For a certain mode of inheritance, x is the number of genes from DV, DL or DU dataset and Non x is the number of genes not from that dataset.

**Data_1** Tables with Entrez Gene ID and degree for each gene belonging to Viable (V), Lethal (L), Disease Viable (DV), Disease Lethal (DL) and Disease

Unknown (DU) dataset.

**Data_2** Table with Gene Ontology number, description and respective viable genes associated wih each GO identifier.

**Data_3** Table with Gene Ontology number, description and respective lethal genes associated wih each GO identifier.

**Data_4** Table with Gene Ontology number, description and respective disease viable genes associated wih each GO identifier.

**Data_5** Table with Gene Ontology number, description and respective disease lethal genes associated wih each GO identifier.

**Data_6** Table with Gene Ontology number, description and respective disease unknown genes associated wih each GO identifier.

**Data_7** Tables with Entrez Gene ID and disease class for each gene belonging to Viable (V), Lethal (L), Disease Viable (DV), Disease Lethal (DL) and Disease Unknown (DU) dataset.

**Data_8** Tables with Entrez Gene ID, associated OMIM ID and disease mechanism for each gene belonging to Viable (V), Lethal (L), Disease Viable (DV), Disease Lethal (DL) and Disease Unknown (DU) dataset.

**Data_9** Tables with Entrez Gene ID and mode of inheritance for each gene belonging to Viable (V), Lethal (L), Disease Viable (DV), Disease Lethal (DL) and Disease Unknown (DU) dataset. AR stands for autosomal recessive, AD stands for autosomal dominant and X represents a gene sex-linked.
